# Retention rate of physicians in public health administration agencies and their career paths in Japan

**DOI:** 10.1186/1472-6963-10-101

**Published:** 2010-04-23

**Authors:** Soichi Koike, Tomoko Kodama, Shinya Matsumoto, Hiroo Ide, Hideo Yasunaga, Tomoaki Imamura

**Affiliations:** 1 Department of Planning, Information and Management, The University of Tokyo Hospital, 7-3-1 Hongo, Bunkyo-ku, Tokyo 113-8655, Japan; 2 Department of Policy Sciences, National Institute of Public Health, 2-3-6 Minami, Wako, Saitama 351-0197, Japan; 3 Department of Health Management and Policy, Graduate School of Medicine, The University of Tokyo, 7-3-1 Hongo, Bunkyo-ku, Tokyo 113-8655, Japan; 4 Department of Public Health, Health Management and Policy, Nara Medical University, 840 Shijo-cho, Kashihara, Nara 634-8521, Japan

## Abstract

**Background:**

Physicians who serve as public health specialists at public health centers and health departments in local or central government have significant roles because of their public health expertise. The aim of this study is to analyze the retention and career paths of such specialists in Japan.

**Method:**

We analyzed the data of seven consecutive surveys, spanning 1994 to 2006. We first analyzed the 2006 survey data by sex, age group, and facility type. We then examined the changes over time in the proportion of physicians working in public health administration agencies. We also examined the distribution of the facility types and specialties in which physicians worked both before beginning and after leaving their jobs. These analyses were performed by using physician registration numbers to cross-link data from two consecutive surveys.

**Results:**

The proportion of physicians working in public health administration agencies was 0.7% in 2006. The actual numbers for each survey ranged between 1,800 and 1,900. The overall rate remaining in public health administration agencies during the two-year survey interval was 72.8% for 1994-1996. The ratio declined to 67.2% for 2004-2006. Among younger physicians with 1-10 years of experience, the retention rate showed a sharp decline, dropping from 72.6% to 50.0%. Many of these physicians came from or left for a hospital position, with the proportion entering academic hospital institutions increasing in recent years. In many cases, physicians left or entered internal medicine clinical practices.

**Conclusion:**

At present in Japan, the number of physicians who leave and the number who begin a position are almost the same; thus, some of the problems associated with physicians leaving are yet to become apparent. However, the fact that the retention period is shortening for younger physicians may represent a future problem for ensuring the quality of physicians in public health administration agencies. Possible strategies include: increasing the number of physicians entering positions; reducing the number leaving positions; and creating a system where physicians can easily reenter positions after leaving while also establishing a revolving door type of career development system, involving both public health departments and hospital clinical departments.

## Background

The roles of public health administration agencies such as public health centers have been broadening. They serve as the front line in implementing infection control measures, including those for new types of influenza, they evaluate and control health risks related food and environmental sanitation, and they take on a coordinating and regulatory role in medical service systems. Among the variety of personnel engaged in these agencies, physicians who serve as public health specialists play significant roles based on their public health expertise, such as a staff member or director of a public health center, or as a public health officer at a department of health in their local or central government. Nonetheless, the quality and career development of these physicians has yet to be sufficiently addressed.

The World Health Organization has published "World Health Organization Global Recommendations for the Retention of Health Workers [1]" and "The World Health Report 2006: Working Together for Health [2]". These reports provide an assessment of the current crisis in the global health workforce. The United States Institute of Medicine publicized "The Future of Public Health" in 1988 and "The Future of the Public's Health in the 21st Century" in 2002. These reports identify retention of the health workforce as the keystone for the infrastructure of public health administration agencies.

In Japan, most physicians in public health administration agencies work at public health centers or in a department of health in their local or national government. During public health emergencies, they are expected to provide impact estimates, establish and carry out proactive policies, and persuade other government organizations, the general public, and management personnel to take action based on their competencies [3]. Japanese public health centers were originally established based on the Public Health Center Law of 1937. Since then, a vast network of health centers has developed, through which all types of preventive services as well as personnel services for local communities are provided [4]. As of 2009, there are 510 public health centers in Japan [5]. A director of public health is required to be a physician with three or more years of public health experience and who has completed a training course at the National Institute of Public Health or its equivalent. However, when it is deemed difficult to recruit a qualified physician, a public health center may hire a non-physician with certain qualifications as a director. The number of such non-physician directors has increased in recent years. In 2004, the national government began the "Project for Promoting the Retention and Registration of Public Health Physicians" to ensure that physicians are retained in public health administration agencies. As of June 29, 2009, 26 physicians have registered with the Ministry of Health, Labour and Welfare (MHLW). Fifty physicians have applied for positions with local governments seeking physicians to serve in the public health field [6].

Although public health physicians form an important part of the public health workforce, little is known about their exact function or educational preparation [7]. The "Committee for Evaluating the Environment for Developing and Retaining Public Health Physicians" was commissioned by Japan's MHLW to study this issue. Under a new clinical training system that began in fiscal year 2004, training programs were introduced at local health and medical care sites. The sites included clinics in remote areas and islands, small and medium-sized hospitals and clinics, public health centers, and welfare facilities. However, evaluations of these training programs have yet to be fully performed.

The purpose of this research is to discuss measures to retain physicians in public health administration agency positions--principally by elucidating their career paths using data from Japan's National Survey of Physicians, Dentists and Pharmacists.

## Methods

### Study design and settings

This study is a retrospective observational study based on an analysis of the National Survey of Physicians, Dentists and Pharmacists conducted by Japan's Ministry of Health, Labour and Welfare. In Japan, all physicians are required to report to the government their status of practice once every two years, pursuant to the Medical Practitioners Law. For the survey, questionnaires were sent from the Ministry of Health, Labour and Welfare to prefectural governments and directly to physicians.

For our current study, we obtained approval from the MHLW to use and analyze survey data collected from 1994 to 2006. The provided data include each physician's registration number, year of obtaining a medical license, sex, age, types of medical services provided, and main area of practice; no data items that could be used to identify individual physicians were included.

### Measures

Two types of measures were used in our analysis: 1) the two-year career continuation rate and 2) the distribution of specialty and facility type for those who had entered or exited from public health administration agencies sometime during their careers.

For the first measure, the two-year continuation ratio, we calculated the ratio of physicians who reported themselves as being employed by a public health administration agency in at least two consecutive surveys among the periods of 1994-1996, 1996-1998, 1998-2000, 2000-2002, 2002-2004, and 2004-2006. For comparison purposes, retention rates for pediatric and obstetrics/gynecology specialists, who have recently attracted attention for their low retention rates, were also calculated.

For the second measure, we calculated the distribution of specialty and facility type to analyze physicians who newly entered public health administration agencies during 2004-2006 and physicians who were employed by a public health administration agency in 2004 but who left the position before 2006. To examine the continuation rate and career path before and after public health administration, two consecutive survey data sets were aggregated using the physicians' registration numbers.

### Samples

The total numbers of physicians who responded to each national survey were as follows: 227,775 (1994); 240,215 (1996); 248,275 (1998); 253,898 (2000); 261,093 (2002); 270,353 (2004); and 277,927 (2006). The estimated reporting rate by existing physicians was about 90% [8]. Those who were working for public health administration agencies were identified by the type of facility indicated in their responses. The characteristics of physicians in public health administration agencies in 2006 are presented in Table 1, and those of physicians in public health administration agencies from 1994-2006 are shown in Table 2.

**Table 1:** Characteristics of physicians in public health administration agencies in 2006

	Physicians in health administration		Total physicians	
		
	Number	%	Number	%
Age distribution				
Under 30	40	2.3%	26,350	9.5%
30-39	235	13.5%	67,057	24.1%
40-49	599	34.4%	70,792	25.5%
50-59	595	34.2%	56,606	20.4%
60-69	223	12.8%	24,930	9.0%
Over 70	49	2.8%	32,192	11.6%
Total	1,741	100.0%	277,927	100.0%
				
Women and their proportions across age groups				
Under 30	17	42.5%	9,428	35.8%
30-39	86	36.6%	16,401	24.5%
40-49	158	26.4%	10,409	14.7%
50-59	146	24.5%	5,903	10.4%
60-69	44	19.7%	2,238	9.0%
Over 70	7	14.3%	3,505	10.9%
Total	458	26.3%	47,884	17.2%
				
Urban/rural distribution				
16 major cities	846	48.6%	88,838	32.0%
Core cities	248	14.2%	43,358	15.6%
Other cities	658	37.8%	127,488	45.9%
Villages and towns	70	4.0%	18,243	6.6%

**Table 2:** Physicians in public health administration agencies from 1994-2006

Year	Number (Female)	% of total Physicians
1994	1,806(463)	0.79%
1996	1,876(457)	0.78%
1998	1,809(467)	0.73%
2000	1,923(491)	0.76%
2002	1,889(473)	0.72%
2004	1,849(488)	0.68%
2006	1,822(484)	0.66%

### Statistical analysis

Chi-square tests were conducted to analyze retention rates and the distribution of facility type and specialties. As for the two-year continuation rate, the null hypothesis that each age group of the 1998-2000 cohort and 2002-2006 cohort groups would show different distributions was tested. We also tested the null hypothesis that the 2004 and 2006 cohorts would show the same distribution for specialty facility type for physicians who had either newly entered or exited public health administration agencies during the 2004-2006 period. SPSS 16.0J (SPSS Japan Inc, Tokyo, Japan) was used for the analysis; p < .01 was considered statistically significant.

## Results

### Current status of physicians employed by public health administration agencies

The number of physicians employed by public health administration agencies in 2006 was 1,822 (0.7% of all physicians). It should be noted that the proportion of those over 40 years old was high, and the proportion of females was also high in all age groups. In addition, with respect to work location, the proportion in urban areas, where many government agencies, including public health administration agencies, are located, was higher (Table 1).

The number of responding physicians for each survey year remained at a fairly constant level, ranging between 1,800 and 1,900, accounting for 0.7-0.8% of all physicians. The proportion of females remained constant at about 25% (Table 2).

### Retention rate of physicians in public health administration agencies

**Figure 1:**
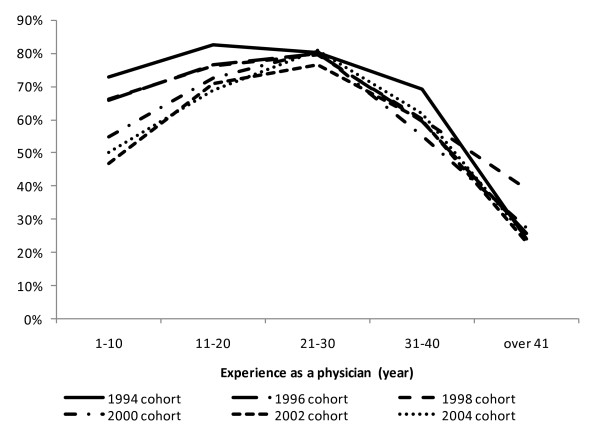
**Retention rates of physicians in public health administration agencies by survey year and age group**. Retention rate for physicians with 1-10 years of experience was 72.8% between 1994 and 1996, but this dropped to 50.0% between 2004 and 2006.

Among physicians employed by public health administration agencies in 2004, the overall proportion of those who remained in the agency two years later, in 2006, was 67.2%. The percentages showed an overall decreasing trend during the 1994-2006 period, from 72.8% to 69.1%, 70.5%, 66.1%, 64.7%, and then to 67.2%. Particularly with younger physicians with 1-10 years of experience, the retention rate declined in every survey year, decreasing from over 70% (72.8%) at the time of the 1994 survey to 50.0% in 2004 (Figure 1). The ratios of those who did not report two years later (no-report ratio) ranged between 10.8% (2004) and 14.1% (2000), showing no significant difference between the surveys.

The retention rates of physicians who specialized in pediatrics or obstetrics/gynecology in the 2004-2006 period were 86.2% and 87.0%, respectively. Regarding physicians with 1-10 years of experience, 81.9% of pediatric physicians and 86.3% of obstetrics/gynecology physicians remained in the same area of practice. The overall no-report ratios were 8.9% and 9.7% for pediatric and obstetrics/gynecology physicians, respectively. Finally, a statistical analysis of the 1998-2000 and 2002-2006 survey group cohorts showed that the groups with 1-10 (p < .001) and 11-20 (p < .001) years of experience had statistically significant lower retention rates for the 2002-2006 survey group.

### Status of physicians' entering and leaving public health administration agency positions

**Figure 2:**
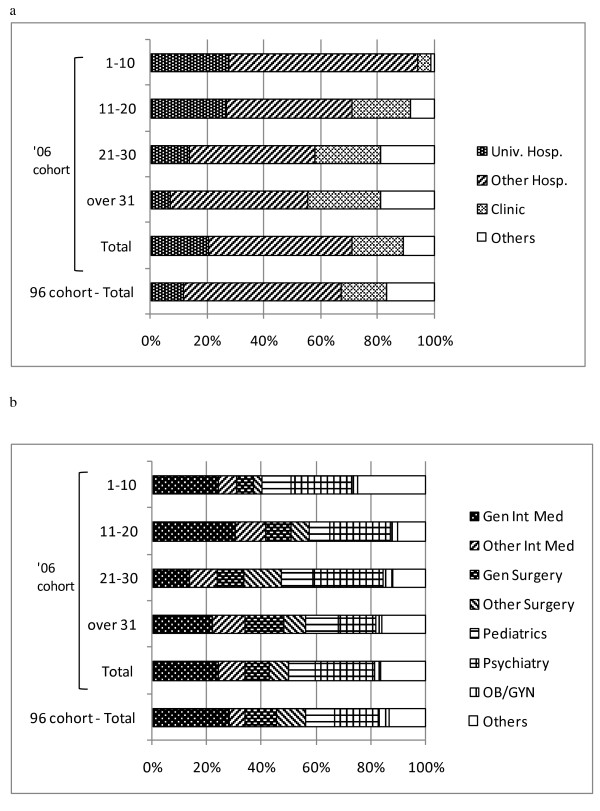
**Previous status of physicians entering into public health administration agencies between 2004 and 2006**. (a) distribution by facility type; (b) distribution by specialty.

With respect to physicians who newly entered public health administration agency positions, we found that many were previously practicing at hospitals. In addition, among the relatively younger physicians (with 1-10 years of experience), the proportion of those who moved from an academic hospital to a public health administration agency was high. Entry into such positions from academic hospitals was found to have increased in recent years. Specifically, an examination of the entries by area of practice revealed that the proportions of entries from the areas of psychiatry and internal medicine were high for all age groups. The proportion of those practicing internal medicine decreased, while that of psychiatry increased, over time (Figure 2). Finally, a statistical analysis of the 1996 and 2006 cohorts revealed a significant difference (p = 0.002) in facility distribution.

**Figure 3:**
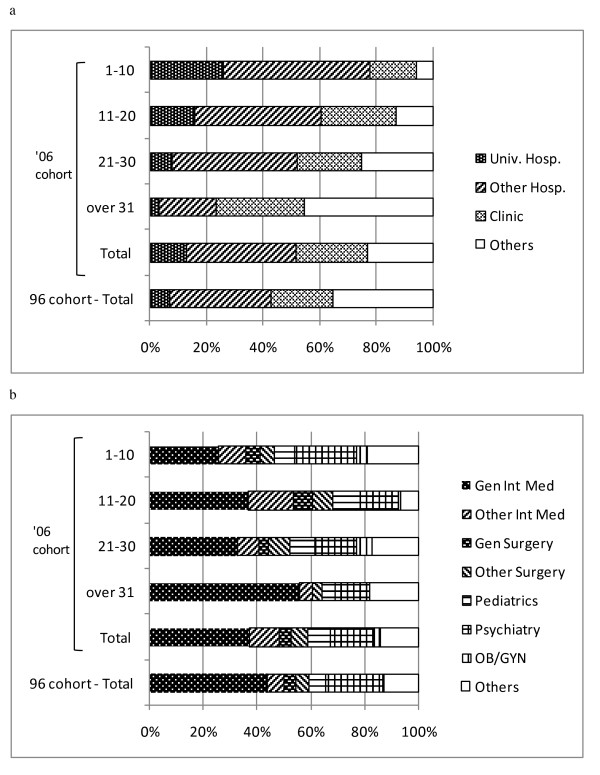
**Exit destination of physicians who left public health administration agencies between 2004 and 2006**. (a) distribution by facility type (b) distribution by specialty.

Of those who left a public health administration agency, many moved to a hospital or clinic. An increased number of younger physicians tended to return to academic hospital positions, and this tendency seems to have increased in recent years. Looking at the position change choices by area of practice, internal medicine positions accounted for almost half, followed by psychiatry positions. Furthermore, among internal medicine physicians, the proportion of those who moved to general internal medicine positions decreased, while those who moved to other internal medicine positions increased. However, the fact that around half of such moves were into internal medicine positions was essentially unchanged (Figure 3). Finally, a statistical analysis of the 1996 and 2006 cohorts revealed significantly different distributions for both the facility distribution (p = 0.002) and the specialty distribution (p = 0.006).

## Discussion

### Number and retention rate of physicians in public health administration agencies

The number of physicians in public health administration agencies ranged from 1,800 to 1,900, accounting for less than 1% of the total number of physicians. Although it is difficult to conduct international comparisons due to variations in the definition of physicians in public health administration agencies, in both Norway [9] and the United Kingdom [10], very few physicians start their careers in public health, and relatively few are employed in public health administration agencies. In the United States, according to a survey conducted by the American Medical Association, although the number of physicians grew from 393,742 to 941,304 between 1975 and 2007, the number of physicians who specialize in public health decreased from 2,665 to 1,436 (-46.1%), and their proportion among all physicians sharply declined from 0.7% to 0.2% during the same period [11]. In Japan, one report showed that the number of physicians in public health centers has decreased [12]. However, because this particular report is focused on public health physicians who serve in prefectural and national government facilities (amongst others), and not those who serve in public health centers, its findings do not contradict our research results.

Our study showed that although the actual numbers of physicians have remained relatively constant, retention rates are decreasing, particularly for younger physicians. For physicians with 1-10 years of experience, the retention rate between 2004 and 2006 was 50%, a sharp decline from 70%, which was the corresponding value between 1994 and 1996. Unlike in the United States, in Japan the number of physicians in public health administration has not decreased. However, physicians do change their posts after only relatively short time spans; this tendency is more prominent among younger physicians. Some experts in Japan have pointed out that there is a particular shortage of physicians in obstetrics/gynecology [13] and pediatrics [14] and this fact, combined with the general shortage of physicians overall, has drawn considerable public attention. Moreover, the proportion of physicians who leave their post within two years is much higher. Meanwhile, because the numbers of physicians who leave and enter positions are almost the same across all public health administration areas, the problem has yet to become apparent. However, from the perspective of ensuring the quality of public health physicians, this is not a favorable situation.

### Reasons for the low retention rate of physicians in public health administration agencies

Because the working conditions for physicians in public health administration agencies are becoming increasingly stressful, the need for risk control measures [15] is increasing. This may partially account for why younger physicians have increasingly left such positions. To address this situation, in 2007 Japan's Ministry of Health, Labour and Welfare commissioned a committee to evaluate the current state of physicians in public health administration agencies; this resulted in a published report, "Review Meeting Report on Evaluating the Environment for Developing and Retaining Public Health Physicians [16]." This report listed reasons for the shortage of physicians in public health administration agencies, including: 1) no long-term plans for recruiting and developing human resources, and lack of human resources management; 2) the appointment of public health center directors only considers the age of candidates, resulting in the assignment of clinicians without experience in public health administration agencies; 3) working in a public health administration agency is not generally seen as an attractive option in which physicians can demonstrate their clinical expertise or in which highly proficient, wide-ranging expert knowledge is required; 4) the ways of entering public health administration agencies are not well known among medical students and physicians interested in public health fields; 5) the available positions are limited, and there is an imbalance of human resources and experience; and 6) there are no collaborative relationships with academic hospitals or other organizations. Thus, this report suggests a need for career development plans for physicians in public health administration agencies.

### Retaining and ensuring the quality of public health physicians

From both the public health and medical strategy points of view, measures to secure the quantity and quality of physicians are greatly needed. In the United States, only 20% of residency graduates practiced in state or local health departments during the 1979-1989 period, a percentage equivalent to the proportion of those who practiced in a nonmilitary branch of the federal government [17]. In addition, almost 80% of top management level officers have not had formal education in public health [18]. A focus-group study in the United States revealed that currently employed public administrators do not have enough time for continuing education [19]. These issues highlight the need for career development of physicians in public health administration agencies.

Our results showed there is a certain career path for physicians entering public health administration agencies. Thus it is important to take a focused approach for each particular physician group, taking into account differences in specialties and facilities. For example, pediatrics and psychiatry are highly represented in public health administration agencies, and they have a relatively close relationship with public health and medical administration agencies. Thus, working with these fields should be effective in quality maintenance and retention.

Possible goals to secure the retention of physicians in public health administration agencies include: increasing the number of physicians entering positions; reducing the number of those leaving positions; and creating a system where physicians who leave a position can return. To that end, it is necessary to communicate the attractiveness of public health administration agencies and to enhance the quality of the existing post-education process. For most physicians, early exposure to public health consisted mainly of clinical medicine, not social medicine. In the Netherlands, for example, although certain sectors of public health attract a high level of interest in the first year of academic hospital placements, the percentage decreases dramatically thereafter; at graduation, public health interest falls well below the average [20]. This may be because the medical curricula pay relatively less attention to public health specialties compared with clinical specialties. Modification of educational curricula, including the CDC-developed Career Path to Public Program (which even targets elementary schools), may be effective in reversing this decline in interest [21].

It is also important to further investigate the possible career paths of physicians in public health administration from the perspective of career development. It may be possible, for example, to build a system that enables the provision of opportunities for those strongly oriented toward clinical practice, even after they have left for a clinical practice position, for example, to later return to public health administration agencies. Other options may include "revolving door" type career paths between the public health departments of medical schools and public health administration agencies. Such efforts would likely help increase the number of physicians at both hospitals and academic hospitals. Furthermore, to broaden the sources of public health physician recruitment, it would serve as a good example if those physicians with work experience in government agencies pursued a successful academic career, thus contributing to the overall development of public health.

### Limitations of this study

This study has several limitations. First, to interpret retention rates in Japan, it should be noted that temporary assignments in different organizations, along with within-organization job rotation, are common practices in Japan. It has been reported that the influence of medical school on graduates remains even after graduation, and thus many junior physicians change their duty station on a rotational basis [22,23]. In such cases, their job category may also change, which often results in their being recorded as having discontinued their public health career; this obviously causes underestimation of the retention rate. Second, because national surveys of physicians, dentists, and pharmacists in Japan only collect static data about where the physicians are currently working, it is impossible to know the reasons why physicians moved from one position to another. Furthermore, more information is needed to determine the actual work situations of physicians, such as whether they work full-time or part-time, or how much they are paid. Third, although the surveys are designed as census surveys, response rates are never 100%. Even if we assume that no response rates are ever distributed equally, this fact may still introduce unpredicted confounding factors into the results. Despite these limitations, however, this study is the first to attempt an investigation of the dynamics of physicians working for public health administration agencies in Japan, and as such, its implications should be applicable to other countries facing the same problem.

## Conclusion

In this study, we analyzed the current status of physicians in the public health sector, the retention rate of those physicians, and their status before and after working in public health administration agencies, by analyzing the data collected in the Survey of Physicians, Dentists and Pharmacists. In Japan, the proportion of physicians in public health administration agencies is less than 1%. Although the number of such physicians has remained at a constant level, the retention rate is declining, especially among younger physicians. Our study revealed that, among physicians with 1-10 years of experience since receiving their medical license, the retention rate has declined to 50%, while that of 10 years ago was over 70%. It is thus necessary to secure the quality of physicians in public health administration agencies.

We suggest that to better retain physicians in public health administration agencies, it is important to undertake initiatives to get physicians more interested in public health. We also suggest that efforts should be made to enhance the quality of current post-education for those already in public health administration agencies, and to develop support systems to meet the needs of a variety of working styles, including responding to the increase in female physicians. Moreover, as jobs in public health administration agencies span a variety of different fields, the importance of career development throughout a physician's working life through "revolving-door" type human resources exchange needs to be recognized.

## Competing interests

The authors declare that they have no competing interests.

## Authors' contributions

SK conceived of the study and participated in the study design, literature review, data analysis, and manuscript drafting. SM, TK, HI, HY and TI participated in the data analysis and manuscript drafting. All authors discussed the results, commented on the manuscript, and gave their final approval.

## Pre-publication history

The pre-publication history for this paper can be accessed here:

http://www.biomedcentral.com/1472-6963/10/101/prepub
